# How to deal with refractory risk factors that depend on behavior?

**DOI:** 10.1192/j.eurpsy.2021.1730

**Published:** 2021-08-13

**Authors:** R. Valido, F. Caldas, P. Ferreira

**Affiliations:** 1 Psychiatry, Hospital de Magalhães Lemos, Porto, Portugal; 2 Internamento C, Hospital de Magalhães Lemos, Porto, Portugal

**Keywords:** behavioral change, behavior, habitual behavior

## Abstract

**Introduction:**

Health-related behavior correlates in critical ways with the current epidemic of chronic diseases. Modifiable behaviors increase the risk of chronic disease. Despite there are well-identified behaviors, efforts at behavior change are clinically-challenging and frequently ineffective.

**Objectives:**

We aim to establish how the current evidence and latest neuroscientific knowledge about behavioral change allow the most reliable assessment of patients with refractory health-related behaviors that negatively impact health outcomes.

**Methods:**

We performed a literature review using Pubmed databases and UpToDate. The search included “behavioral change” and “health-related behavioral change”[MeSH Terms].

**Results:**

Habitual behavior consists of behavioral patterns operating below conscious awareness and acquired through context-dependent repetition. Behavioral change is a complex multi-level field of intervention. The Health Belief Model allows a careful description of the patient’s perceived vulnerability, perceived disease severity, self-efficacy, and change motivation. The identification of social variables is critical since they correlated with poor health outcomes, particularly in chronic diseases. Temperament and character traits can have a strong influence on the difficulty of changing habitual behavior. Psychopathology, if present, must be addressed because it can be a notable factor of behavior instability and correlates negatively to health outcomes. Assertive and efficient communication skills in the clinical context are imperative. Motivational interviewing skills can allow effective behavioral change.

**Conclusions:**

Interventions addressing behavior change require careful, thoughtful work that leads to a deep understanding of the nature of what motivates people. Intervention based strategies focused on behavioral change must undergo further investigation in the future.

**Disclosure:**

No significant relationships.

